# Bioprosthetic Valve Fracture After TAVR Complicated by Balloon Rupture: Bail-Out TAVR in TAVR in SAVR

**DOI:** 10.1016/j.jaccas.2022.07.038

**Published:** 2022-10-05

**Authors:** Tiziana Attisano, Michele Bellino, Francesco Vigorito, Antongiulio Maione, Amelia Ravera, Adele Pierri, Cesare Baldi, Gennaro Galasso, Carmine Vecchione, Raoul Bonan

**Affiliations:** a Cardio-Thorax-Vascular Department, University Hospital San Giovanni di Dio e Ruggi d’Aragona, Salerno, Italy; b Department of Medicine, Surgery and Dentistry, University of Salerno, Baronissi, Italy; c Vascular Physiopathology Unit, Department of Angio-Cardio-Neurology, IRCCS Neuromed, Pozzilli, Italy; d Institut de Cardiologie de Montreal, Faculty of Medicine, University of Montreal, Montreal, Quebec, Canada

**Keywords:** aortic valve, complication, echocardiography, valve repair, valve replacement, BVF, bioprosthetic valve fracture, EOA, effective orifice valve area, PPM, patients-prosthesis mismatch, SAVR, surgical aortic valve replacement, TAVR, transcatheter aortic valve replacement, THV, transcatheter heart valve, ViV, valve-in-valve

## Abstract

Transcatheter aortic valve replacement in surgical aortic valve is a safe and effective procedure to treat patients with failed bioprosthetic surgical valves at high risk for reoperation. Performing bioprosthetic valve fracture has been shown to improve postprocedural hemodynamics of TAVR in surgical aortic valve replacement. However, specific complications related to valve fracture are becoming more common. (**Level of Difficulty: Advanced.**)

## Central Illustration

**Figure:**
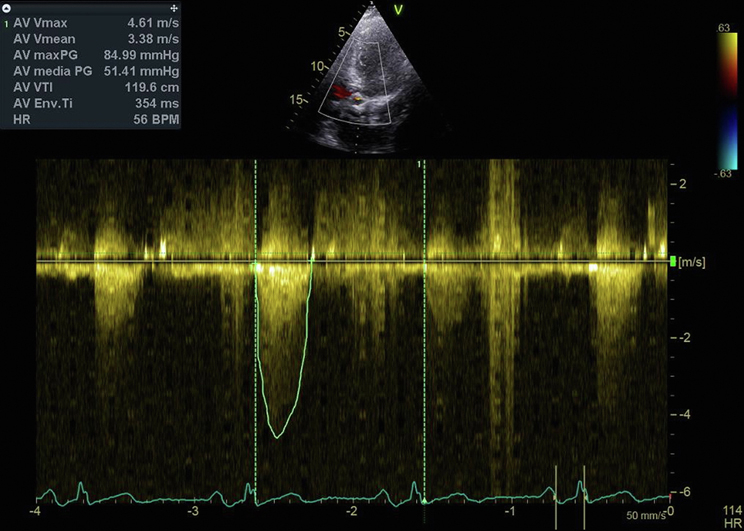

## History of Presentation

A 61-year-old male patient with dyspnea at rest and asthenia was admitted to our department. Physical examination revealed tachycardia at 110 beats/min, a blood pressure of 110/70 mm Hg. The patient had a body surface area of 1.84 m^2^/kg. A holosystolic murmur grade 3 followed by a proto diastolic murmur was heard. Chest auscultation reveled basal bilateral pulmonary rales. Electrocardiography documented sinus rhythm with left bundle branch block.

**Learning Objectives**

• To treat patients with degenerated surgical bioprosthesis and high surgical risk through ViV TAVR, a safe and effective procedure.
• To safely perform BVF in experienced centers with reduction of high residual gradients following ViV TAVR due to PPM.
• To avoid PPM at the time of index SAVR, especially in younger patients.
• To better understand balloon reliability at high pressure during BVF, with the support of further research and experience.

## Past Medical History

His past medical history was characterized by coronary artery bypass graft in 2006 and surgical aortic valve replacement (SAVR) with a 21-mm bioprosthesis in 2017. The patient also carried treated high blood pressure, dyslipidemia, chronic kidney disease stage 3a, and chronic obstructive pulmonary disease stage B.

## Investigation

**Figure 1:**
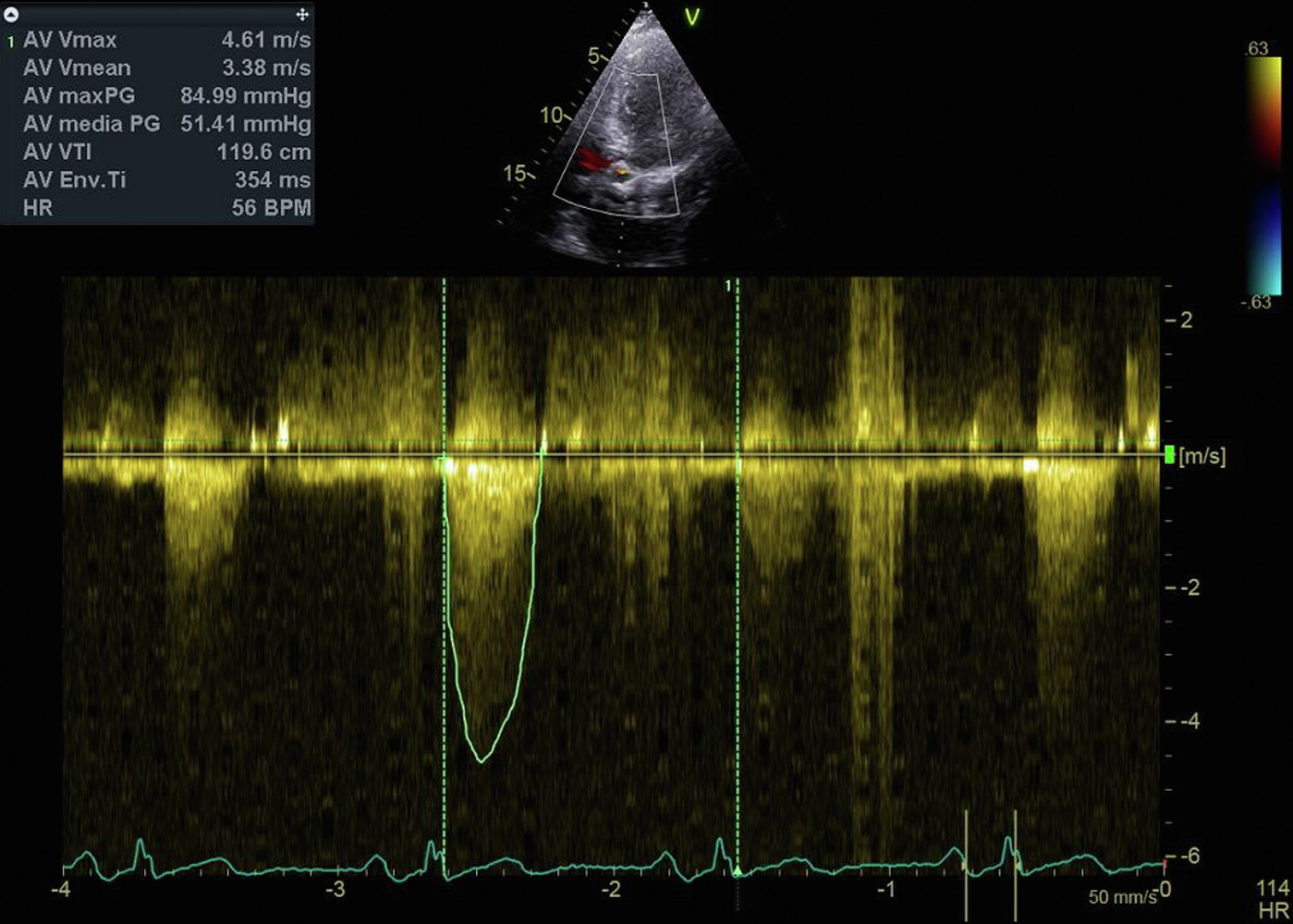
Continuous Doppler on Aortic Bioprosthesis Continuous Doppler shows a mean gradient of 51.41 mm Hg. AV = atrioventricular; BPM = beats/min; HR = heart rate; VTI = velocity time integral.

Transthoracic echocardiography showed a severe bioprosthesis valve degeneration with a mean transvalvular gradient of 51 mm Hg and indexed effective orifice valve area (EOA) of 0.5 cm^2^/m^2^ with moderate regurgitation (Figure 1). Transthoracic echocardiography also documented left ventricle enlargement (end-diastolic volume of 96 mL/m^2^), severe reduction of the ejection fraction (33%) with a stroke volume of 22 mL/m^2^ and an estimated pulmonary arterial systolic pressure of 50 mm Hg. His B-type natriuretic peptide level was 2,765 ng/mL. The third stage of chronic kidney injury was also confirmed. STS-PROM was calculated of 9.2%.

Once the clinical condition was stabilized, the patient underwent coronary angiography which excluded new significative coronary lesions and documented all bypass graft patency.^1^

Invasive transaortic pressure gradient confirmed a mean gradient of 60 mm Hg with a derived EOA of 0.5 cm^2^/m^2^. The patient was evaluated by the heart team to decide between reoperation vs valve-in-valve (ViV) transcatheter aortic valve replacement (TAVR).

**Figure 2:**
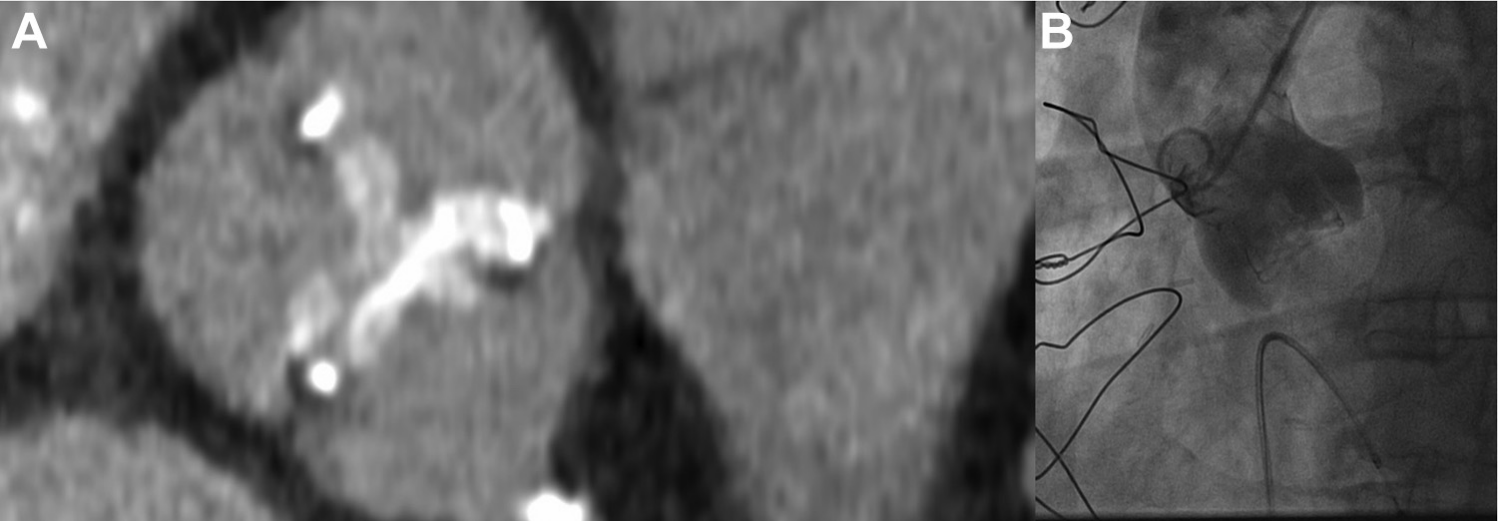
Computed Tomography and Angiographic Images Showing Relationship Between Coronary Ostia, Surgical Bioprosthesis and Aorta **(A)** Transverse plane at the level of the post of the aortic bioprosthesis obtained from the angiographic computed tomography. Image shows a correct alignment of the post of surgical valve and coronary ostium. The distance between coronary ostia and valve was >9 mm, indicative of a low probability of coronary obstruction during valve-in-valve intervention. **(B)** Aortography performed before the valve-in-valve procedure.

**Figure 3:**
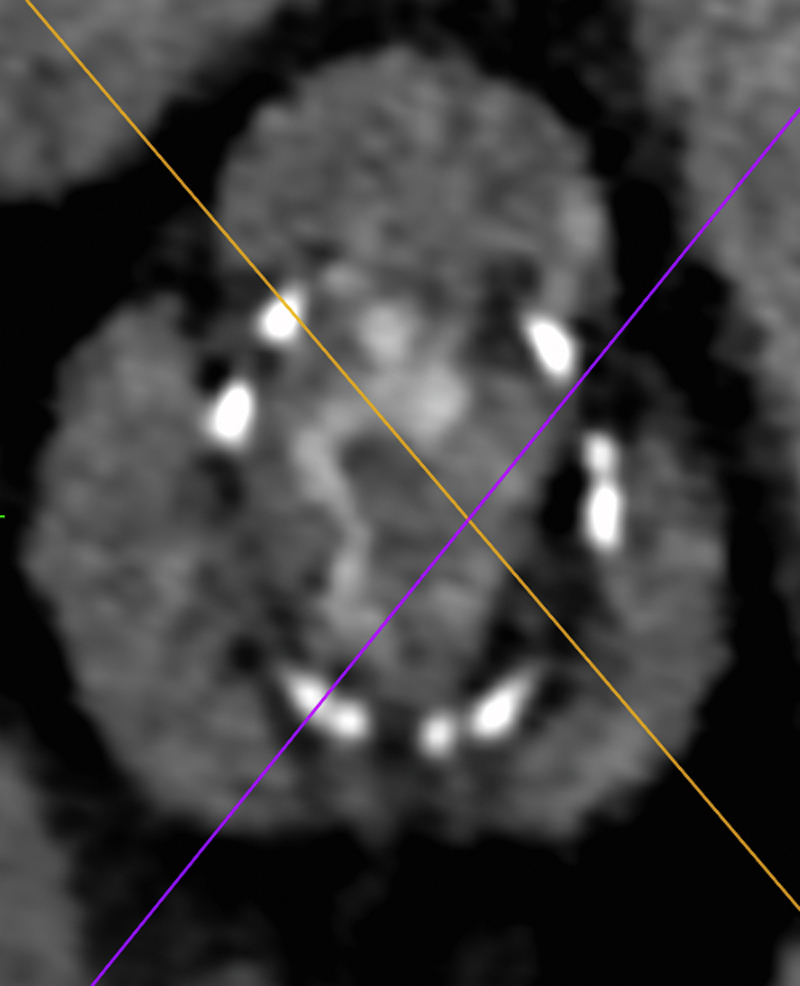
Computed Tomography Image at the Plane of Failed Surgical Bioprosthesis Transverse plane at the level of valve’s annulus showing that coronary ostia were not affected by surgical bioprosthesis encumbrance and testifying a very low risk of coronary occlusion.

Computed tomography demonstrated adequate femoral access for TAVR, valve-to-coronary distance >9 mm, and a Sino-tubular junction diameter of 25 mm (Figures 2 and 3).

## Management

**Figure 4:**
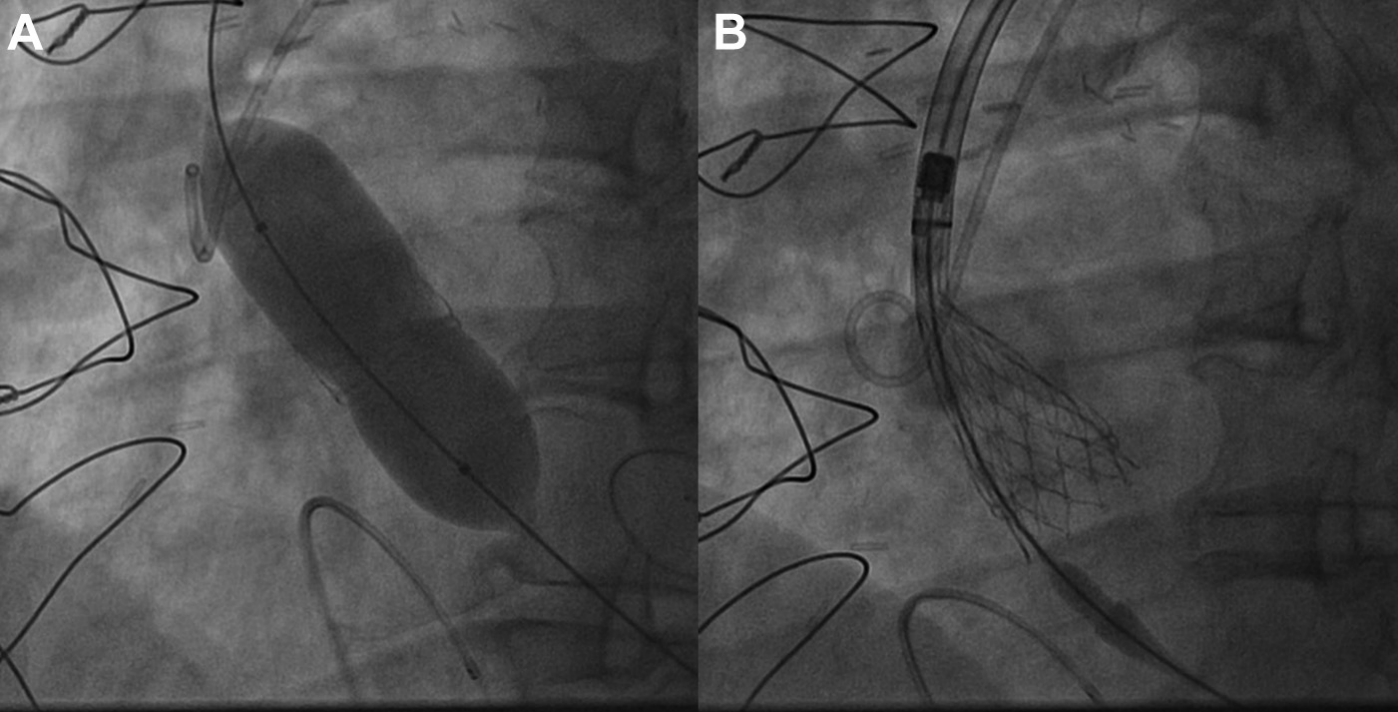
Valve-in-Vale Intervention With the Deployment of a Percutaneous 23-mm Valve in a Surgical 21-mm Valve **(A)** Predilatation of failed surgical aortic bioprosthesis; **(B)** percutaneous 23-mm valve deployment.

**Figure 5:**
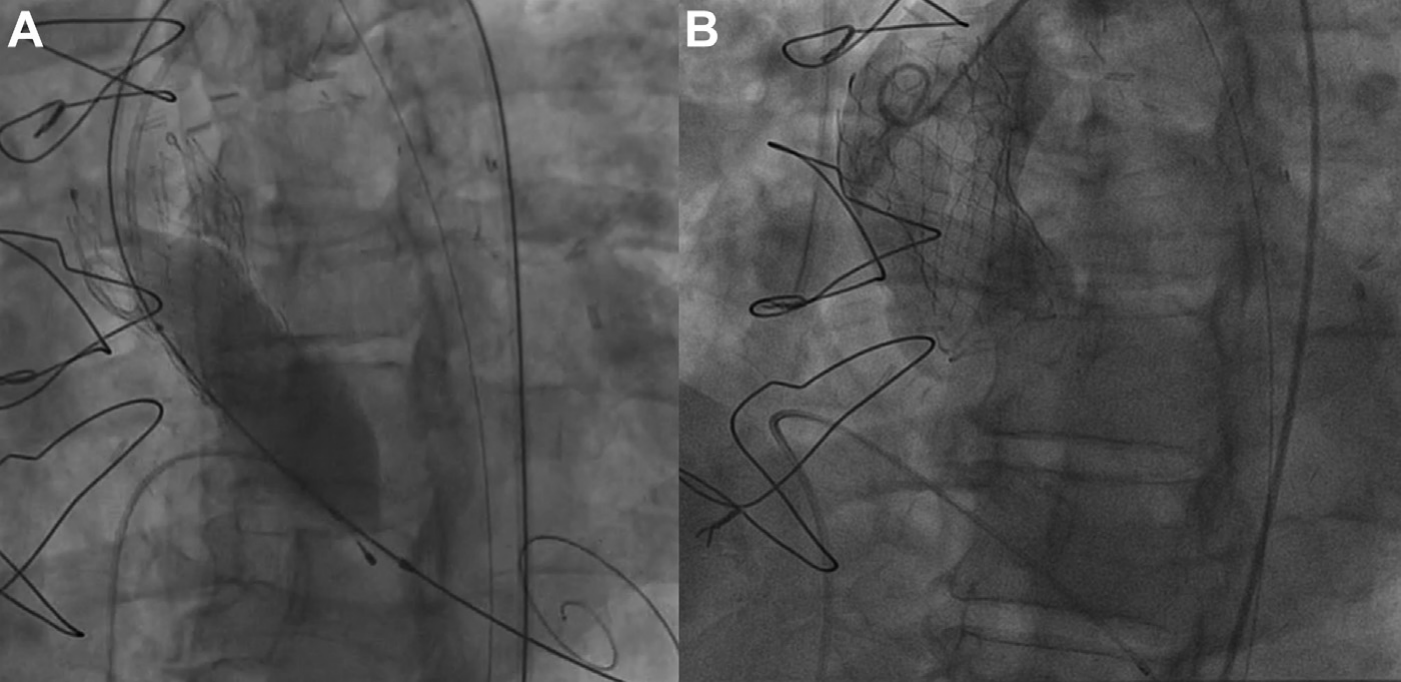
Bioprosthetic Valve Fracture Complicated by Balloon Rupture and Entrapment Followed by Newly Implanted Valve Dislocation **(A)** Bioprosthetic valve fracture resulting in rupture and entrapment of the balloon. **(B)** Anterograde displacement of the newly implanted bioprosthesis in ascending aorta.

**Figure 6:**
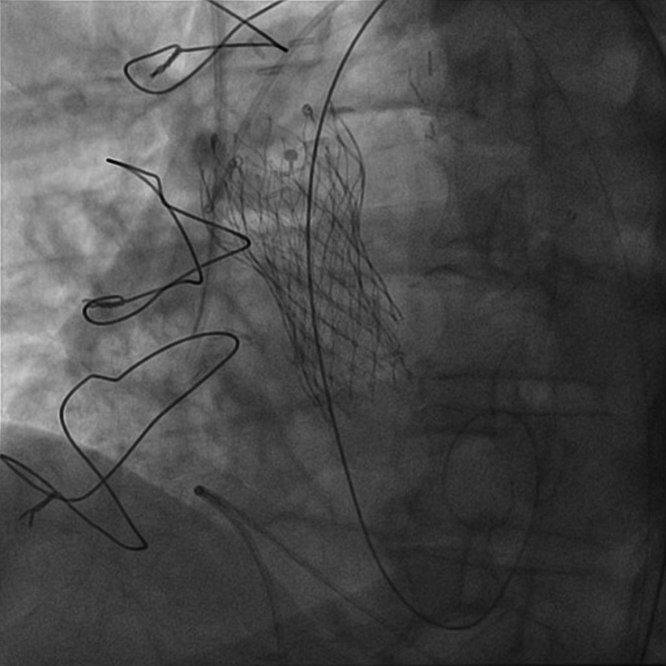
Bail-out Implantation of a Second Percutaneous 23-mm Valve in the Displaced Valve (Valve-in-Valve-in-Surgical Aortic Valve Replacement Procedure)

The heart team recommended ViV TAVR using a supra-annular self-expandible 23-mm valve. The transaortic valve was rightly implanted through femoral access (Figure 4), but a residual mean transvalvular gradient of 31 mm Hg was documented. The decision was made to perform bioprosthetic valve fracture (BVF). A 22-mm noncompliant balloon was inflated to 26 atm, as recommended for patient’s valve type.^2^ A visible release of the balloon waist was obtained on fluoroscopy, indicating that the bioprosthetic ring had been fractured.^3^ As described in the bench side model, for the patient’s valve type, the mechanism of fracture was probably caused by a linear dissection of the outer ring of the bioprosthesis attributable to a single fracture line, the latter not clearly viewable at angiographic control. Unfortunately, at the end of the inflation, despite an expected balloon burst pressure of 30 atm, the balloon ruptured and was trapped through the just-implanted valve frame^2^ (Video 1). After several attempts to remove the trapped balloon, the last decisive maneuver, although effective, resulted in anterograde ascension of the newly implanted valve clearing the coronary ostia (Figure 5). The patient’s clinical condition rapidly worsened with cardiogenic shock and need for mechanical ventilation in relation with a severe aortic valve regurgitation. On the wire kept in place, a second percutaneous 23-mm valve (TAVR-in-TAVR-in-SAVR) was promptly implanted in perfect position, leading to excellent hemodynamics (Figure 6). A mean transvalvular gradient of 7 mm Hg, in the absence of any aortic regurgitation, was recorded, suggestive for effective BVF as recently reported in the literature.^4^ The patient was extubated before transferring to the cardiological intensive care unit. Postprocedure hospitalization was uneventful. After the procedure, left ventricular stroke volume was 32 mL/m^2^, and an indexed EOA of 0.70 cm^2^/m^2^ was calculated.

## Discussion

We presented the first case, to our knowledge, of a ViV TAVR done through BVF of a degenerated 21-mm surgical aortic bioprosthesis followed by displacement of the first implanted percutaneous valve and massive aortic regurgitation caused by rupture and entrapment of balloon at the end of inflation at 26 atm, treated by bail-out implantation of a second 23-mm percutaneous valve (valve-in-valve-in-valve). Surgical bioprosthetic valve degeneration is a well-known condition with reoperation rates of ≈10% and 30% at 10 and 15 years, respectively.^5^

The ViV procedure has emerged as a novel option whereby a transcatheter heart valve (THV) is implanted within a failed surgical heart valve. However, some concerns arise, mostly regarding patient-prosthesis mismatch (PPM), particularly in patients with small bioprosthetic valves (≤21 mm) associated with reduced survival.^1^ In the VIVID registry, 32% of patients had severe PPM following VIV TAVR, which has been associated with increased long-term mortality following both surgical and THV implantation.^5^

BVF emerged as a novel technique to address this problem in targeted types of surgical bioprosthesis. According to the bench tests, the BVF procedure appears to be effective for sewing ring fracture if a dedicated pressure of inflation is reached.^1^ In a large multicenter series, BVF was safely performed along with both balloon and self-expanding THVs, resulting in significantly lower transvalvular gradients and increased EOA. In addition, 1-year follow-up after BVF demonstrates persistent low gradients, no signal for TAVR injury, and improved survival compared with historical control subjects.^6^

As reported by recent metanalysis, after ViV-TAVR performed with BVF, the difference in means for mean valve gradients showed a significant reduction (random-effects model: −26.7; −28.8 to −24.7; *P <* 0.001), whereas the difference in means for aortic valve area showed a significant increase (random-effects model: 0.55 cm^2^; 0.13-0.97; *P =* 0.029).

However, despite the improvement in aortic valve area means, these remain too low (<1.5 cm^2^) after the procedure, highly likely caused by the small size of the bioprosthetic valves implanted during the index SAVR. This should be kept in mind, also considering an ever-growing number of patients with obesity. Even when we change the thresholds for PPM for obese patients, as suggested by some authors, the risk of PPM is very high.^7^ Consequently, the index SAVR acquires great importance in avoiding PPM.^8^ If surgeons are unable to implant an appropriate valve, they should resort to surgical techniques to enlarge the aortic annulus, making sure that the patient will receive a large valve and will not leave with PPM.^9^

Furthermore, it should be kept in mind that PPM is not only caused by the size of the valve chosen during the index SAVR but also ascribed to patient's BSA, and this must be considered on a case-by-case basis.^10^

It should also be considered that a series of complications with BVF have been described. Particularly, a case of balloon rupture without clinical adverse event has been reported.^3^ In our case, after BVF performed with a 22-mm balloon caused by unacceptable residual gradient, the balloon remained entrapped in the frame of the bioprosthesis resulting in its anterograde displacement during the removal phase. Further research and experience are needed to better understand the really and safety reliability of balloon at high pressure.

### Follow-up

**Figure 7:**
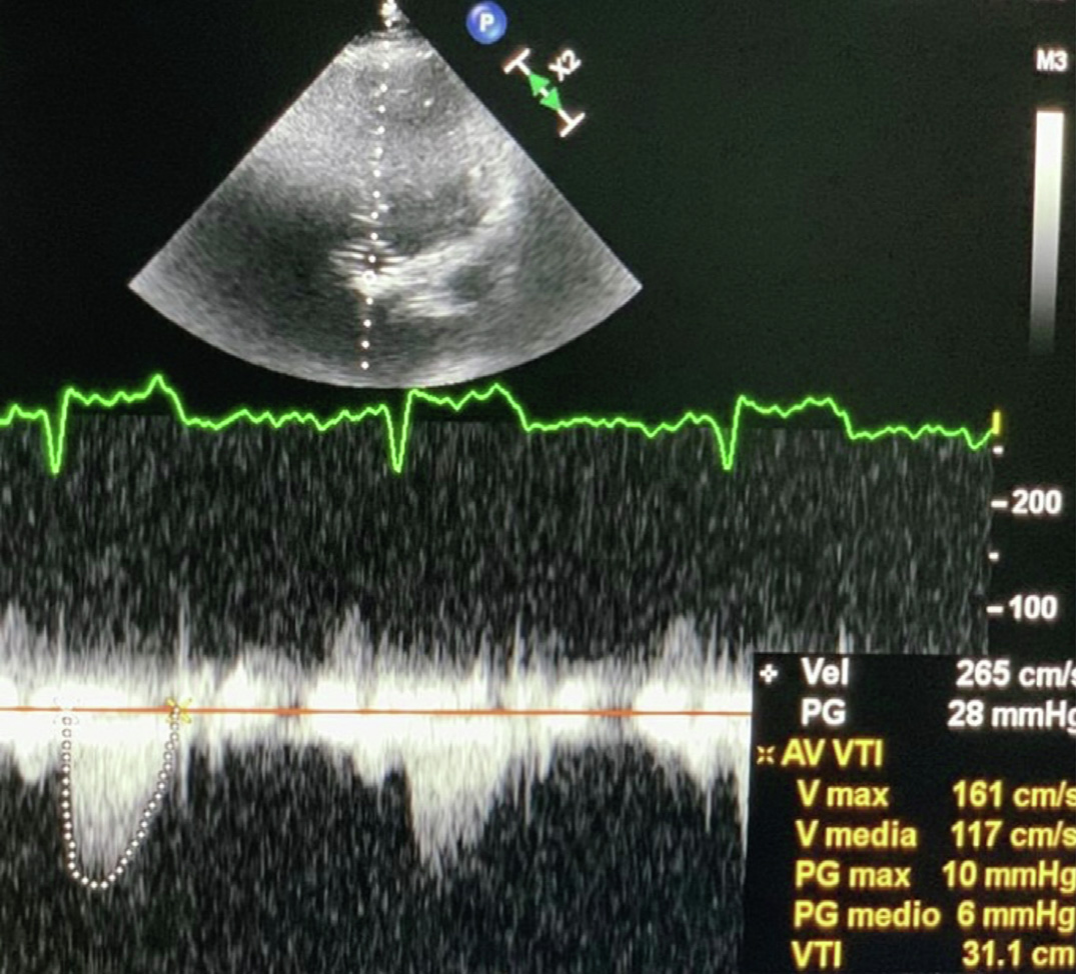
Continuous Doppler Through the Inner Aortic Bioprosthesis at 1-Year Follow-up Showing Normal Mean Gradient PG = pressure gradient; other abbreviations as in Figure 1.

At 1-year follow-up, the patient was asymptomatic, with a mean transvalvular gradient of 8 mm Hg, in the absence of valve regurgitation or paravalvular leaks (Figure 7). EOA and indexed EOA of the implanted aortic valve were calculated of 1.61 cm^2^ and 0.88 cm^2^/m^2^, respectively.

## Conclusions

BVF is a novel technique conceived to reduce gradients in VIV-TAVR procedures by fracturing the sewing ring of the bioprosthesis through high-pressure noncompliant balloon inflation. BVF can be performed safely but with caution and expertise, achieving reduction of high residual gradients following ViV-TAVR. Whether all surgical valves, regardless of size and residual gradients, should be fractured to optimize transcatheter valve expansion is currently being debated.

## Funding Support and Author Disclosures

The authors have reported that they have no relationships relevant to the contents of this paper to disclose.

## Appendix

Supplemental Video 1 A 22-mm Noncompliant Balloon Inflated to 26 atm, as Recommended for Patient’s Surgical Valve Type, to Obtain BVF Through the First Percutaneous Implanted Valve. A visible release of the balloon waist was obtained on fluoroscopy, but inflation was followed by balloon rupture and entrapment in valvular frame.
